# The Role of Chronic Stress as a Trigger for the Alzheimer Disease Continuum

**DOI:** 10.3389/fnagi.2020.561504

**Published:** 2020-10-22

**Authors:** Marina Ávila-Villanueva, Jaime Gómez-Ramírez, Fernando Maestú, César Venero, Jesús Ávila, Miguel A. Fernández-Blázquez

**Affiliations:** ^1^Alzheimer Disease Research Unit, CIEN Foundation, Carlos III Institute of Health, Queen Sofía Foundation Alzheimer Center, Madrid, Spain; ^2^Laboratory of Cognitive and Computational Neuroscience (UCM-UPM), Center for Biomedical Technology, Campus de Montegancedo, Madrid, Spain; ^3^Department of Experimental Psychology, Complutense University of Madrid (UCM), Campus de Somosaguas, Madrid, Spain; ^4^Department of Psychobiology, Universidad Nacional de Educación a Distancia (UNED), Madrid, Spain; ^5^Center of Molecular Biology Severo Ochoa (CSIC-UAM), Campus de Cantoblanco, Madrid, Spain; ^6^Network Center for Biomedical Research in Neurodegenerative Diseases (CIBERNED), Madrid, Spain

**Keywords:** aging, Alzheimer disease, dementia, mild cognitive impairment, stress, subjective cognitive decline

## Introduction

Alzheimer disease (AD) is the most prevalent type of dementia that appears late in life and has devastating effects both in society and patients. This is a silent disorder in which neurodegeneration occurs in the brain decades before the diagnosis of the disease (Bateman et al., 2012). This long period of time between the appearance of the first pathophysiological changes and the presentation of clinical symptoms suggests that there is an AD continuum in which different transition stages can be distinguished. Thus, prior to the onset of dementia, it appears a prodromal stage called mild cognitive impairment (MCI) which is characterized by the presence of cognitive deficits, but not severe enough to significantly affect everyday activities (Petersen et al., 1999). In addition, earlier in the continuum and before the appearance of MCI, a preclinical AD phase has been described. This preclinical stage is defined by the accumulation of biomarkers in the brain as well as the appearance of a state termed subjective cognitive decline (SCD). SCD is defined as the presence of self-reported subtle cognitive complaints despite normal performance in standard cognitive testing (Jessen et al., 2014). The difficulty in pharmacologically modifying the course of AD continuum has fostered the consensus that therapeutic interventions are more likely to be effective at the earliest possible phase. Then, early markers are urgently needed to identify how the silent neurodegeneration is taking place before the onset of clinical signs of dementia. For this purpose many candidates have been proposed so far. In the present manuscript we would like to highlight the role of stress, a much less explored risk factor for AD.

## Description of the Stress Response

Stress may affect multiple neural pathways and brain systems in different ways and at different times. Firstly, stress elicits a very rapid response on the brain. The amygdala activates the hypothalamus and brainstem by increasing dopaminergic and noradrenergic activity and altering PFC functioning (Arnsten, 2009). Activation of the sympathetic nervous system leads to the release of peripheral catecholamines adrenaline and noradrenaline from the adrenal medulla which prepare the organism for a fight-or-flight response (Korte et al., 2005). Subsequently, on a timescale of about 10–15 min, stress also activates the hypothalamic-pituitary-adrenal (HPA) axis. The HPA is a major neuroendocrine system that controls reaction to stress through the production of cortisol, the most important glucocorticoid in humans, which can be measured in saliva in just 25 min (Kudielka et al., 2004); cortisol is able to cross the blood-brain barrier to bind neural receptors located on the hippocampus, the amygdala and the prefrontal cortex (Lupien and McEwen, 1997; de Kloet et al., 1999). The final body response to the chronic stress involves the immune system through the production of pro-inflammatory cytokines, which can directly influence neural activity in the brain (Harrison et al., 2015).

## Influence of Stress on Aging and Neural Substrates

There is evidence that chronic stress can accelerate aging that is the main risk factor for AD. The ability of aged organisms to generate an adequate stress response decreases compared to younger organisms (Fonken et al., 2018). During aging, the functioning of cell glucocorticoid receptors decreases and free (toxic) cortisol can arise, leading to damage cerebral areas. Also, aging can promote inflammatory priming through a process involving microglia activation and changes in circadian rhythms via a mechanism including cortisol signaling (Fonken et al., 2018). This signaling is disrupted in human aging resulting in an altered circadian rhythm as indicated above and, as a snake that bites its own tail, sleep disturbances may cause an increase in cortisol secretion (Schouten et al., 2019). Moreover, cognitive and affective neural networks can be altered during aging due to the possible link between both systems and adrenal medulla, the last component of HPA axis (Canet et al., 2019; Konishi et al., 2019). Chronic stress via dysregulation of the HPA-axis can be a trigger of co-morbid depression in neurodegenerative diseases (Rapp et al., 2011; Du and Pang, 2015). Finally, chronic stress have been reported to accelerate AD pathogenesis in mouse models for AD, including extracellular beta-amyloid plaque deposition and intracellular tau hyperphosphorylation (Carroll et al., 2011; Sotiropoulos et al., 2011; Justice et al., 2015). The exacerbation of both AD hallmarks may be due, at least in part, to excessive secretion of corticosteroids, as it has been reported that corticosteroids injection alone may raise deposits of beta-amyloid plaques and fibrillary tangles (Wang et al., 2011; Joshi et al., 2012). However, it is likely that the excess of corticosteroids is not the only mechanism by which stress exacerbates AD neuropathology, since manipulations of the neuropeptide released by stress Corticotropin Releasing Factor can also alter beta-amyloid release and tau aggregation (Justice, 2018).

The hippocampus, a region that plays a key role in memory encoding and retrieval, is the brain structure most associated with AD so that hippocampal atrophy is considered the gold standard brain biomarker. In the hippocampus, cortisol receptors, both glucocorticoid (GR) and mineralocorticoid (MR), are present (de Kloet et al., 1999). High-affinity MR appear to have a protective role and promote resilience, whereas low-affinity GR may play a role in promoting neuronal death; the balance between both types of receptors is advisable for a proper hippocampal function (Rogalska, 2010; Yau et al., 2011). Stress is capable of breaking the balance between both receptors, leading to a loss of thickness in the hippocampus. A recent study examined the pattern of atrophy in rats' hippocampal subfields under physical and psychological stressors (Li et al., 2019). Under both conditions atrophy was first identified in CA1 mainly associated with physical stress, while CA3 and dentate gyrus were affected later. Interestingly, when physical stress disappeared, the brain effects could progressively revert to normal, but atrophy of the dentate gyrus did continue to shrink even though psychological stress had ceased (Bai et al., 2012; Li et al., 2019). Other animal studies have provided direct evidencet of the deleterous effects of glucocorticoids on hippocampal morphology. Thus, sustained exposure to elevated corticosteroid levels has been found to alter dendritic morphology and reduce hippocampal volume in different hippocampal subfields (Woolley et al., 1990; Sousa et al., 1998, 2000). More recently, it has been observed that old rats submitted to long-term social isolation, a strong psychological stress situation, leads to increased plasmatic corticosterone levels and to a specific reduction in the volume of the *stratum oriens* and spine density in CA1 that occurred concomitantly with impairment in spatial memory (Pereda-Pérez et al., 2019). In humans, although the evidence in older adults is somehow inconclusive (Cox et al., 2017), a similar pattern of hippocampal atrophy largely consistent with animal models has been described. Thus, older adults with persistently high cortisol levels over a 5-year period, or even with higher levels of perceived chronic stress, show a preferential volume loss in CA4/dentate gyrus and CA2/CA3 subfields (Lupien et al., 1998, 2007; Zimmerman et al., 2016). It is precisely the atrophy in the dentate gyrus associated with stress in animals and humans that may be the key; this region plays a critical role in the sustained neurogenesis throughout adult life (Epp et al., 2013). Adult neurogenesis has been observed in most mammals, including humans (Schoenfeld and Gould, 2012), and currently it is well-known that neurogenesis is related to cognitive impairment and dementia (Moreno-Jiménez et al., 2019).

High levels of cortisol may also be present in Cushing's syndrome. Patients with this condition show an increase of cortisol levels in blood, being the body exposed to high levels of that hormone during a long time. Cortisol affects primarily to peripheral tissues, but it can also affect brain structures secondarily leading to a cognitive impairment (Forget et al., 2000; Frimodt-Møller et al., 2019). However, despite Cushing's syndrome could add some evidence on the relationship between cortisol and cognitive impairment, this model does not seem to be totally comparable with neurodegenerative disorders since patients with Cushing tend to show a more premature mortality than sporadic AD patients (Dekkers et al., 2007; Clayton et al., 2016).

Chronic stress in midlife could cause a dysregulation in that balance leading to a malfunctioning of the hippocampus in the long-term. Therefore, people more prone to psychological distress face a higher risk for MCI (Wilson et al., 2007). As previously indicated, this objective cognitive decline could appear after the SCD stage (Ávila-Villanueva et al., 2016, 2018; Ávila-Villanueva and Fernández-Blázquez, 2017). Analyzing people with SCD, it was found that such individuals contained higher levels of salivary cortisol, the surrogate of stress (Fiocco et al., 2006; Peavy et al., 2013).

## Discussion

**Figure 1:**
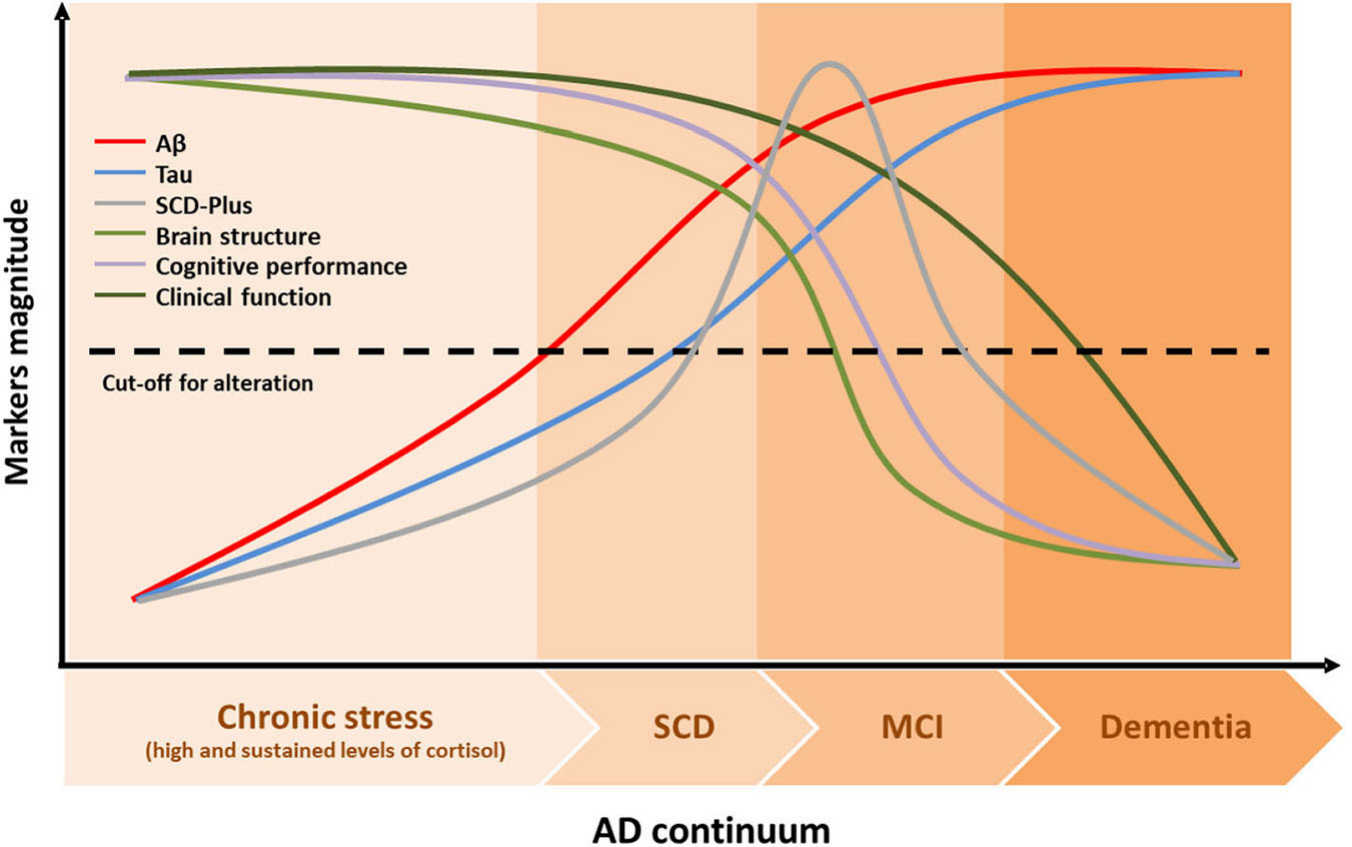
Theoretical dynamic of AD continuum. The AD pathological cascade model embodied the following assumptions in the temporal dynamic of the disease: (1) biomarkers become abnormal in a temporally ordered manner as the disease progresses; (2) Aβ identified by CSF Aβ42 or PET amyloid imaging occurs early in the disease, long time before the appearance of clinical symptoms; (3) tau-mediated neuronal injury identified by CSF appears later in the disease spectrum; (4) brain changes captured by structural MRI are the last biomarker to become abnormal; however, those brain changes would retain a closer relationship with cognitive performance than other biomarkers; and (5) cognitive and functional deterioration are the last symptoms that appear in this model. We hypothesize that there would be a third curve namely SCD-Plus that would occur after tau deposition, but just before brain structure starts changing, in the preclinical AD stage. Chronic stress defined with elevated and sustained cortisol levels over time could precede and act as a trigger of the AD pathological cascade. Aβ, β-amyloid; AD, Alzheimer's Disease; MCI, Mild Cognitive Impairment; SCD, Subjective Cognitive Decline.

Looking for possible connections between stress and the AD continuum, salivary cortisol levels-that reflects the levels of biologically active, free cortisol in serum-seem to be a particularly promising marker for cognitive decline. It is well-documented that stress may affect the memory systems and the ability to remember past events (Schwabe et al., 2012). While acute stress is somewhat adaptive and may have beneficial effects on memory functioning in specific situations (Yuen et al., 2009; Shields et al., 2017), chronic stress is associated with a variety of alterations through the production of glucocorticoids, specifically cortisol, that could play a role in decreasing memory encoding and consolidation (Csernansky et al., 2006; Peavy et al., 2007). Thus, based on available evidence, it can be suspected a link between cortisol levels and the progression of dementia. An increase in the levels of cortisol in MCI subjects has been previously found (Venero et al., 2013) and recently confirmed in an article published in Frontiers in Aging Neuroscience (Ho et al., 2020). Additionally, it has been suggested that individuals with SCD have increased levels of salivary cortisol (Fiocco et al., 2006; Peavy et al., 2013). The analysis of salivary cortisol could, therefore, become a suitable marker for the diagnosis of MCI or AD. Thus, looking for earlier markers to know which feature could be recognize first during the silent development of AD, we would like to suggest the chronic stress suffered especially in midlife and measured by the level of salivary cortisol, may precede and act as a trigger of SCD that could appear before MCI, which can take place before the early clinical symptoms of AD (Figure 1). To confirm that hypothesis, we postulate that longitudinal studies to analyze the transition from a non-demented stage to SCD and MCI stages should include along with molecular biomarkers (i.e., beta-amyloid and tau) the measurement of salivary cortisol, as proposed by Ho et al. (2020) to confirm, or not, that the order of events prior to development of AD is that indicated in Figure 1.

## Author Contributions

All authors are responsible for the conceptualization, reviewing the literature, and critically editing the manuscript. All authors approve the submitted version of the manuscript and are accountable for the accuracy and integrity of the work.

## Conflict of Interest

The authors declare that the research was conducted in the absence of any commercial or financial relationships that could be construed as a potential conflict of interest.
